# Characterization of induced pluripotent stem cell‐derived megakaryocyte lysates for potential regenerative applications

**DOI:** 10.1111/jcmm.13698

**Published:** 2018-06-12

**Authors:** Anja Baigger, Dorothee Eicke, Yuliia Yuzefovych, Denys Pogozhykh, Rainer Blasczyk, Constanca Figueiredo

**Affiliations:** ^1^ Institute for Transfusion Medicine Hannover Medical School Hannover Germany

**Keywords:** growth factors, megakaryocytes, platelet lysate, regenerative medicine

## Abstract

Recently, platelet‐derived growth factors present in lysates became an extreme interest in the field of regenerative medicine such as in wound healing and as substitutes to foetal bovine serum in xeno‐free cell culture systems. However, the generation of such platelet lysates completely depends on the availability of platelet donors. In this study, the possibility to use *in vitro*‐generated megakaryocytes derived from induced pluripotent stem cells (iPSCs) as a cell source for typical platelet growth factors was investigated. Therefore, the presence and levels of those factors were characterized in *in vitro*‐produced megakaryocytes. In comparison with platelets, *in vitro*‐generated megakaryocytes showed a multifold increased content in transcript and protein levels of typical platelet growth factors including platelet‐derived growth factors (PDGFs), transforming growth factor (TGF)‐1β, vascular endothelial cell factor (VEGF)‐A, epidermal growth factor (EGF), insulin‐like growth factor (IGF)‐1 and tissue factor (TF). Hence, iPSC‐derived megakaryocytes may serve as an efficient cell source for a donor‐independent generation of growth factor‐rich lysates with a broad application potential in innovative cell culture systems and regenerative therapies.

## 1 INTRODUCTION

Megakaryocytes (MKs) are bone marrow residential platelet (PLT) precursors which release their progeny by cytoplasmic shedding into the sinusoidal vasculature. PLTs play key roles in the regulation of haemostasis and wound healing.^1^ Sprouting PLTs are equipped with a plethora of PLT‐typical growth factors (GFs) and bioactive molecules of megakaryocytic origin^2^ which essentially mediate migration, expansion and differentiation of cells into the wound region. These natural PLT characteristics can be exploited by the generation of PLT‐derived products, such as PLT lysates (hPL). GF‐rich hPLs were considered of extreme relevance in regenerative medicine and stem cell therapies^3^ as well as substitutes to foetal calf serum (FCS) in xeno‐free cell culture systems.^4^ Mainly platelet‐derived growth factor (PDGF), transforming growth factor (TGF)‐1β, vascular endothelial growth factor (VEGF)‐A, epidermal growth factor (EGF), insulin‐like growth factor (IGF)‐1 and tissue factor (TF) have been described as relevant active factors contributing for the regenerative potential of hPLs.^3^, ^4^ Due to their immunomodulatory, pro‐mitogenic/angiogenic and wound healing‐supporting properties, hPLs are of clinical interest as adjuvant therapies for the treatment of ulcers,^5^ ocular lesions or graft versus host disease 6 such as for joint and bone regeneration.^7^

However, the translational perspective of hPL is impaired by limitations in the availability of PLT donors.^8^ Here, the in vitro pharming of MKs and PLTs from induced pluripotent stem cells (iPSCs) opens new perspectives for a donor‐independent generation of lysates. In contrast to other cell sources such as cord blood or mobilized haematopoietic progenitor cells, iPSC is readily available in sufficient numbers to the large‐scale production of MKs. The generation of iPSC‐derived PLTs has been confronted with technical challenges which significantly limit their production in large numbers. In contrast, MKs can be efficiently differentiated in vitro from iPSCs in a functional stage and large numbers, while saving time and costs for the final differentiation steps from MKs to PLTs.^9^ Therefore, in this study, we have investigated whether the known factors responsible for the regenerative properties of hPL are already present in iPSC‐derived MKs and their lysates. In vitro‐generated MKs might serve as an alternative to hPL. Therefore, this study aimed to establish a protocol for the generation of plasma‐free MK lysate (MKL) and to determine the presence of PLT‐typical GFs at transcript and at protein level in MKL and furthermore the potential to support cell culture.

## 2 MATERIALS AND METHODS

### 2.1 Megakaryocyte enrichment and the production of lysates

Megakaryocyte differentiation, morphological and phenotypic analysis were performed as previously described in Börger et al. (2016).^10^ MKs were enriched from the differentiating cultures using anti‐CD61‐specific microbeads (Miltenyi Biotech) and magnetic‐activated cell sorting (MACS, Miltenyi Biotech). MKLs were generated by exposure of the CD61^+^‐MKs to six cycles of freezing and thawing, followed by centrifugation for 60 minutes at 13 000 rpm and 4°C. Lysates were then filtrated through 0.22‐μm ultralow protein‐binding filters.

### 2.2 Analysis of growth factors transcript levels

Total RNA isolation was performed using the RNA easy Kit (Qiagen, Hilden, Germany), according to the manufacturer's instructions. Reverse transcription was carried out using the high‐capacity cDNA reverse transcription kit (Applied Biosystems, Darmstadt, Germany). Transcript levels were analysed by qRT‐PCR using GF‐specific TaqMan gene expression assays (Thermo Fisher Scientific, Braunschweig, Germany).

### 2.3 Quantification of growth factors protein levels

IGF‐1 and TF levels were measured using R&D Systems Quantikine ELISAs (Minneapolis, MN). PDGF‐AA, PDGF‐BB, PDGF‐AB and VEGF‐A levels were determined using ELISAs purchased from Thermo Fisher Scientific. TGF‐β1 and EGF were analysed by ELISAs from Invitrogen (by Thermo Fisher Scientific). All assays were performed according to the manufacturer's instructions. ELISA measurements were normalized to the total protein content of the lysates.

### 2.4 Proliferation assay with mesenchymal stromal cells

To evaluate the efficacy of MKL in supporting the growth of bone marrow mesenchymal stromal cells (bmMSCs), proliferation assays were performed. bmMSC were isolated as described previously.^11^ Briefly, bone marrow aspirates were obtained during routine orthopaedic procedures from the healthy donors by iliac crest aspiration. Human bmMSCs were isolated from fresh heparinized aspirates with density gradient centrifugation and subsequent recovery of mononuclear cells. For proliferation assays, bmMSCs were labelled with eBioscience™ Cell Proliferation Dye eFluor™ 670 (CPD, Thermo Fisher Scientific) and seeded in 96‐well culture plates, at a density of 5000 cells/well in 100 μL basal medium (DMEM, 1% sodium pyruvate, 1% penicillin/streptavidin) without supplement or supplemented with either 10% FCS, 10% hPL, 10% MKL or 20% MKL. After 3 days of culture, cell morphology was analysed by light microscopy. Number of viable cells was determined using the sensitive colorimetric assay “Cell Counting Kit—8” (CCK‐8, Sigma Aldrich). CPD staining was analysed by flow cytometric analysis to monitor cell proliferation.

### 2.5 Statistical analyses

All data were analysed using GraphPad Prism 5 software (GraphPad Software, San Diego, CA). Statistical analyses were performed using two‐tailed *t* tests. Levels of significance were expressed as *P*‐values (*P ≤* *.05, **P ≤* *.01 and ***P ≤* *.001).

## 3 RESULTS AND DISCUSSION

**Figure 1:**
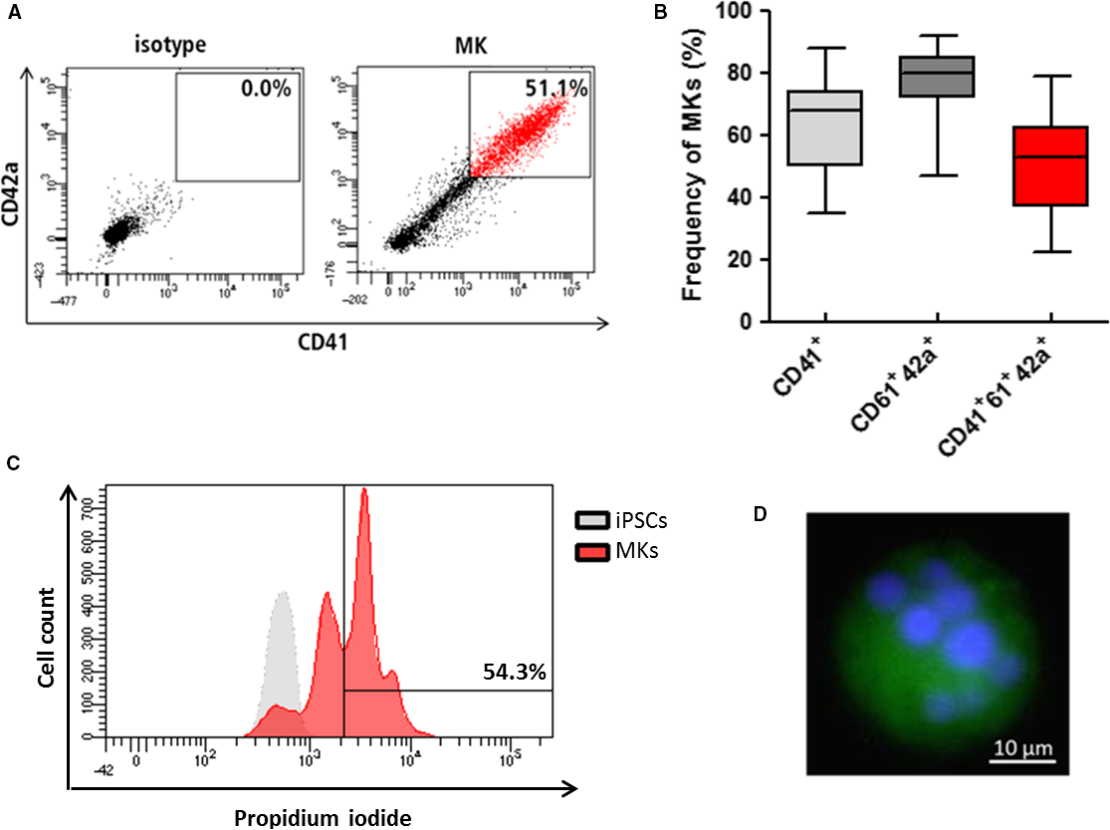
Phenotypic and morphological characteristics of in vitro‐generated MKs. After 22 days of differentiation from iPSC, in vitro MKs were analysed for MK typical features. A, MKs were identified by the expression of the typical markers CD41 and CD42a as indicated in the representative dot plot. B, In vitro‐differentiated cells revealed high frequencies of MKs. Box depicts median, upper and lower percentile. Whiskers depict minimum and maximum, n = 15. C, Representative FACS histogram revealing an increase in DNA content. MKs were gated as CD41^+^ cell population from which the PI content was depicted. IPSC was used as a reference for diploidy. D, In vitro MKs showed polyploid nuclei in fluorescence microscopy analyses after staining using anti‐CD61 antibody (green) and DAPI (blue)

While an evaluation of natural MKs for transfusion medicine or novel regenerative purposes is critically impaired by difficulties to access bone marrow and the low abundance of MKs within bone marrow, the in vitro production of MKs enables to exploit their potential in the development of potential therapeutic strategies. Previously, we have shown that MKs can be effectively differentiated from iPSCs under xeno‐free and defined conditions 10 and in large scale.^9^ Also in this study, we have differentiated MKs from iPSCs. Flow cytometric analyses revealed the presence of MKs in the differentiation cultures (Figure 1A). On day 22, harvested cell populations show in average 50.09% ± 15.01% CD41^+^CD61^+^CD42a^+^‐positive MKs (Figure 1B) with typical polyploid nuclei (Figure 1D) and increased DNA content (Figure 1C).

**Figure 2:**
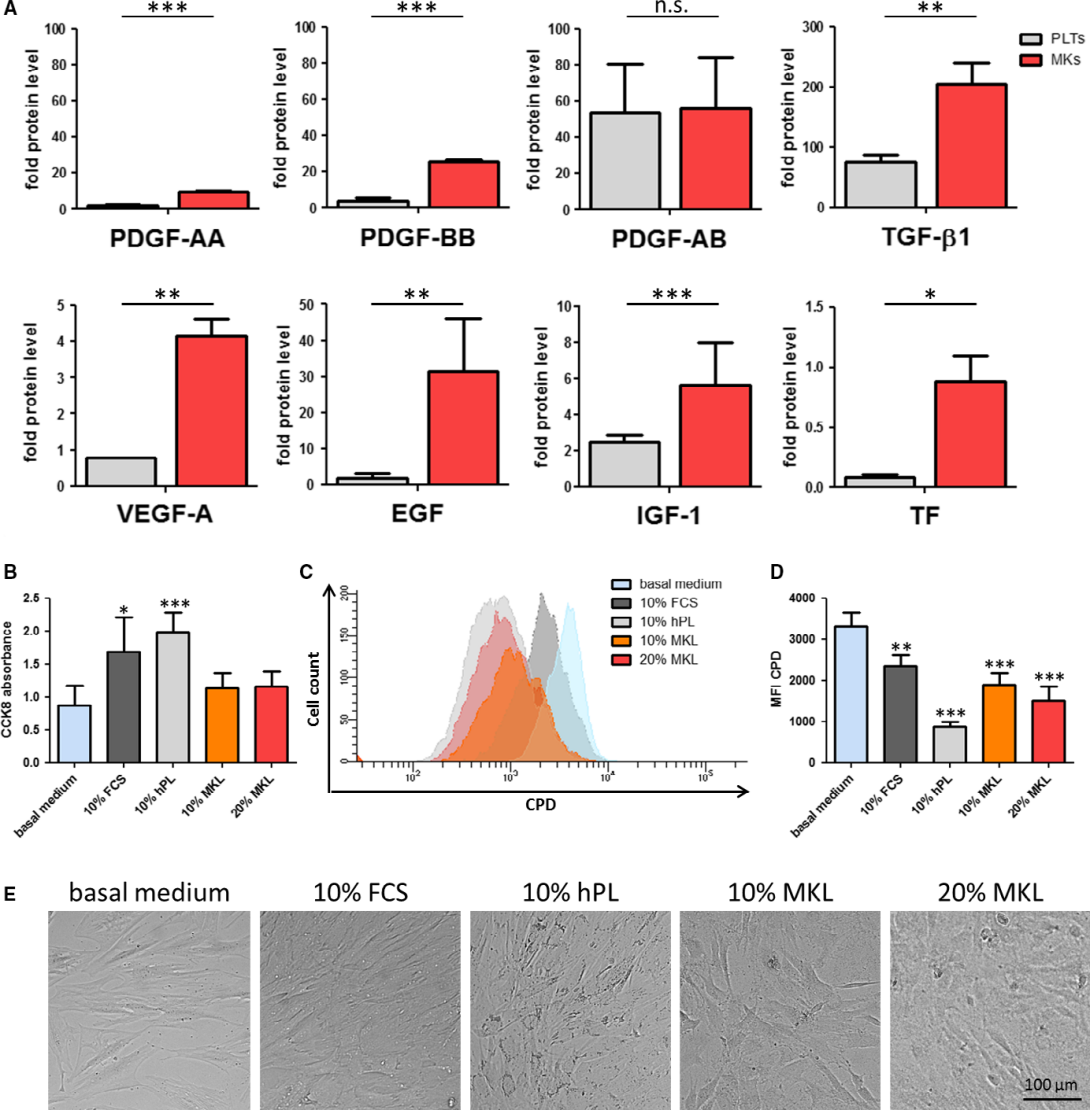
MKL shows increased levels of PLT‐typical GFs and improve cell proliferation. A, The presence of GFs in MKL and hPL at protein level was analysed and compared by ELISA. MKLs reveal significantly higher content of EGF, IGF‐1, PDGF‐AA, PDGF‐BB, TF, TGF‐β1 and VEGF‐A; and similar levels of PDGF‐AB compared to standard hPL. Graph depicts means ± SD, n = 4. ***P ≤ .001, **P ≤ .01, *P ≤ .05; n.s. not significant. MSCs were labelled with CPD and cultivated in basal medium without supplement, with 10% FCS, 10% hPL, 10% MKL or 20% MKL. B, The amount of viable cells was analysed using CCK8 assay. Cell proliferation was analysed after 3 days of culture by flow cytometry. Exemplary overlay histogram (C) and means (D) are shown. Graphs depict mean ± SD, n = 5. E, Light microscopy pictures of the cultures show density and morphology of the bmMSCs

Analyses of the MKL proteomic content showed that PLT‐typical GFs were detectable (Figure 2A and Table S2). These GFs were previously shown to play pivotal roles in regenerative medicine, in particular, PDGF hetero‐ and homodimers (PDGF‐AA, PDGF‐BB, PDGF‐AB) with relevant functions in wound healing. Likewise, in vitro iPSC‐derived MKs exhibit TGF‐β1 which has previously shown to act synergistically to PDGFs in wound healing. Moreover, we also could detect GFs associated with angiogenesis and epithelialization, such as VEGF and EGF. TF, the major key driver in blood coagulation, and IGF‐1 which serves as a mitogen for diverse healing capable cells from mesenchymal origin were also detectable in iPSC‐derived MKs.^12^, ^13^

Combined with the analysis of the GF transcript levels (Figure S1 and Table S1), this reflects an active translation of those GFs in in vitro iPSC‐derived MKs as well as preservation of high GF levels throughout the lysate manufacture. Several clinical studies yet demonstrated that topical application of PDGF‐BB, EGF or VEGF accelerates the healing of chronic ulcers.^12^ In comparison with hPL, in vitro‐generated MKL demonstrates a combination and high contents of such stimulatory GFs, indicating MKL might be suited for an application in in vitro culture systems, tissue remodelling and angiogenesis.

For the generation of hPL, conventionally donor‐derived PLTs are lysed in plasma. However, plasma‐reduced or plasma‐free formulations of hPL have been established,^4^ which circumvent potential downsides related to plasma contained isoagglutinins, coagulation factors and infections. Remarkably, plasma‐free MKLs demonstrated significantly higher levels of GFs than conventional hPL (Figure 2A). Only PDGF‐AB levels were found to be disproportional compared to the expression levels of the other GFs in MKL (Figure 2A). Intriguingly, other approaches intentionally deplete PDGF and VEGF from hPL, as these GFs are implicated in cancer development 13 without reducing the proliferative effect of hPL. Hence, no negative functional aspects in MKL are expected due to decreased PDGF‐AB ratio in comparison with other GFs. In addition, we have detected the presence of angiogenesis‐related miRNAs such as miR‐16, miR‐21 and miR‐126 in MKL at significantly higher levels than in hPL (Figure S2).

The applicability of hPL as an alternative to FCS during the in vitro expansion of bmMSC has been extensively evaluated.^14^ In this study, we showed that MKL as supplement to basal cell culture medium supports viability and proliferation of bmMSC (Figure 2B‐D). We have detected an increase in the number of viable bmMSC when MKL was used in comparison with the basal medium, but not as strong as with FCS or hPL. Nevertheless, MKL induced higher proliferation of bmMSCs than FCS (Figure 2C and D). All different conditions appear to affect differently the morphology of bmMSCs (Figure 2E). Hence, MKL showed to modulate bmMSC survival and proliferative capacity, confirming the presence of bioactive factors. Further studies will be required to determine the concentration of MK per lysate to achieve a maximal effect. However, the distinct origin of FCS, hPL and MKL associated with their distinct proteomic content is expected to contribute to different effects of FCS, hPL and MKL on bmMSCs. Here, future studies combining proteomic profiling and functional tests of hPL and MKL will be essential to identify the essential set or minimum levels of GFs required to support cell culture systems 15 and specific therapeutic effects.

Despite the potential clinical use of iPSC‐derived cell products remains controversial mainly due to the associated tumorigenic risk, the cell‐free MKL would pose significantly lower safety concerns. In addition, MKL safety could also be increased by gamma irradiation. Furthermore, the therapeutic efficiency of MKL might be modulated by genetically engineering of the iPSC line used for MK production to overexpress desirable growth factors or regulatory molecules as well as to knock down the expression of potential immunogenic proteins such as HLA or human platelet antigens.^8^

Altogether, our data indicate that in vitro‐manufactured iPSC‐derived MKs may serve as a suitable cell source for the generation of GF‐rich secondary cell products, such as whole cell MKLs. Current advances in the generation of large‐scale in vitro culture systems for the production of 2 × 10^8^ MK from iPSCs in only 50 mL scale 9 will permit an unlimited, cost‐ and time‐effective production of MKL. Hence, this study supports the establishment of next‐generation in vitro stimulants for innovative research and therapeutic applications.

## CONFLICT OF INTERESTS

The authors have no conflict of interests.
