# Supplementary figures and images for: Characterization of induced pluripotent stem cell‐derived megakaryocyte lysates for potential regenerative applications
Source: J Cell Mol Med. 2018 Jun 12;22(9):4545–9. doi: 10.1111/jcmm.13698 (PMC6111809; doi:10.1111/jcmm.13698)

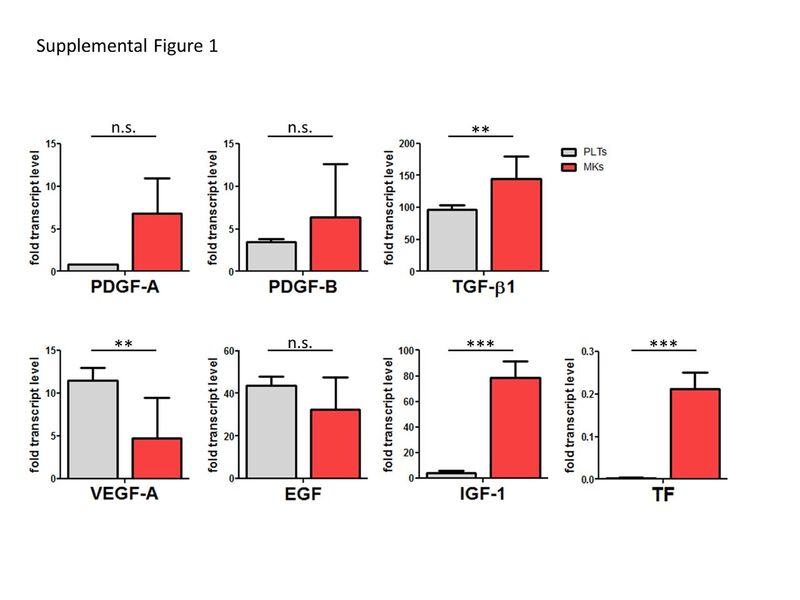

Supplement: Supplementary file 1 [file JCMM-22-4545-s001.jpeg]

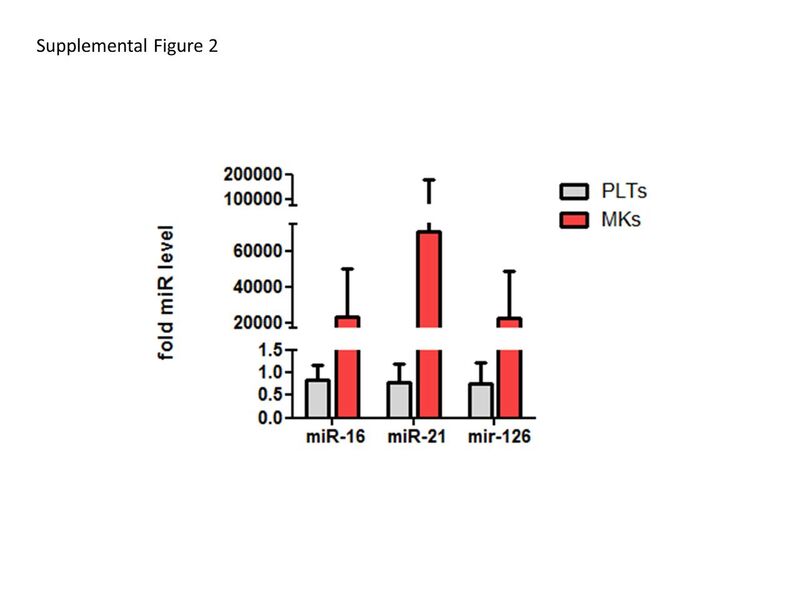

Supplement: Supplementary file 2 [file JCMM-22-4545-s002.jpeg]
