# Supplementary material for: Characterization of induced pluripotent stem cell‐derived megakaryocyte lysates for potential regenerative applications
Source: J Cell Mol Med. 2018 Jun 12;22(9):4545–9. doi: 10.1111/jcmm.13698 (PMC6111809; doi:10.1111/jcmm.13698)
Supplement: Supplementary file 3 [file JCMM-22-4545-s003.docx]

# Supplemental information

# Materials and Methods

*Analysis of growth factors transcript levels*

Total RNA isolation was performed using the RNA easy Kit (Qiagen, Hilden, Germany), according to the manufacturer’s instructions. Reverse transcription was carried out using the high-capacity cDNA reverse transcription kit (Applied Biosystems, Darmstadt, Germany). Transcript levels were analyzed by qRT-PCR using GF specific Taqman gene expression assays (Thermo Fisher Scientific, Braunschweig, Germany).

*Analysis of angiogenesis related microRNAs*

To test for the presence of specific miRNAs, total RNA was isolated from MKLs and hPL using the miRNeasy Serum/Plasma Kit (Qiagen). To normalize the measurements during the later RT-PCR analysis, a synthetic *C. elegans* miR-39 miRNA mimic (Qiagen) was added as an internal spike-in control after the addition of QIAzol. Reverse transcription was performed according to the manufacturer’s instructions of the miScript II RT Kit (Qiagen).

# Results

Accordingly to the protein levels of the PLT-typical GFs, also the transcripts of these factors were detectable (Supplem. Fig 1 and Supplm. Table 1). Furthermore, the presence of miRNA related to angiogenesis was shown. The levels of miR-16, miR-21, and miR-126 were significantly elevated in comparison to hPL (Supplem. Fig 2).

# Figure legends and Tables

Supplemental Figure 1

GF transcript levels of donated PLTs and *in vitro* generated MKs were analyzed by qRT-PCR. Transcript levels are shown as the fold increase normalized to iPSC level. Donated PLTs and *in vitro* generated MKs reveal transcripts for PLT-typical GF. Statistical difference was considered to be significant at p ≤ 0.05 (**p<0.01, ***.p<0.001, n.s. not significant).

Supplemental Figure 2

Levels of miR-16, miR-21, and miR-126 in hPL and MKL were analyzed by RT-PCR. The values, normalized to an artificial control are represented as mean ± SD (n=3). Statistical difference was considered to be significant at p ≤ 0.05 (***p<0.001).

Table 1. Growth factors transcript levels in iPSC-derived MKs and donated PLTs.

The fold increase in MK or PLT transcript levels was compared to iPSC-level and is represented by its mean ± SD (n=4). Also, the ratio of MK/PLT transcript level for each GF as well as p-values are provided. Statistical difference was considered to be significant at p ≤ 0.05.

| Transcript level | **MK** | **PLT** | **MK/PLT** | **p-value** |
| --- | --- | --- | --- | --- |
| **EGF** | 32.61 ± 14.84 | 43.75 ± 4.25 | 0.75 | 0.133 |
| **IGF-1** | 78.73 ± 12.84 | 4.31 ± 1.51 | 18.26 | 0.001 |
| **PDGF-A** | 6.80 ± 4.11 | 0.86 ± 0.01 | 7.90 | 0.272 |
| **PDGF-B** | 6.34 ± 6.24 | 3.52 ± 0.27 | 1.80 | 0.241 |
| **TF** | 0.21 ± 0.04 | 0.003 ± 0.00 | 69.10 | 0.002 |
| **TGF-β1** | 144.49 ± 35.00 | 96.94 ± 7.12 | 1.49 | 0.006 |
| **VEGF-A** | 4.75 ± 4.75 | 11.51 ± 1.51 | 0.41 | 0.005 |

Table 2. Growth factors protein levels in iPSC-derived MKL and conventional hPL.

For MKL and hPL the fold increase at protein level normalized to iPSC-level is given by mean ± SD, next to the ratio of MKL/hPL protein level and the p-value between MKL and hPL levels. Statistical difference was considered to be significant at p ≤ 0.05. Table lists means ± SD, n = 4.

| Protein  level | **MKL** | **hPL** | **MKL/hPL** | **p-value** |
| --- | --- | --- | --- | --- |
| **EGF** | 31.32 ± 14.75 | 1.93 ± 1.29 | 16.20 | 0.004 |
| **IGF-1** | 5.63 ± 2.34 | 2.46 ± 0.45 | 2.29 | 0.020 |
| **PDGF-AA** | 9.88 ± 0.27 | 2.29 ± 0.12 | 4.31 | 0.000 |
| **PDGF-AB** | 69.93 ± 1.39 | 53.37 ± 26.66 | 1.31 | 0.193 |
| **PDGF-BB** | 25.16 ± 1.77 | 3.99 ± 1.32 | 6.31 | 0.000 |
| **TF** | 0.88 ± 0.21 | 0.09 ± 0.02 | 10.19 | 0.000 |
| **TGF-β1** | 205.19 ± 35.66 | 75.69 ± 12.21 | 2.71 | 0.003 |
| **VEGF-A** | 3.76 ± 0.39 | 0.77 ± 0.02 | 4.86 | 0.006 |
